# Androgen Insensitivity Syndrome: Clinical Phenotype and Molecular Analysis in a Single Tertiary Center Cohort

**DOI:** 10.4274/jcrpe.galenos.2018.2018.0185

**Published:** 2019-02-20

**Authors:** Maria Sol Touzon, Natalia Perez Garrido, Roxana Marino, Pablo Ramirez, Mariana Costanzo, Gabriela Guercio, Esperanza Berensztein, Marco A. Rivarola, Alicia Belgorosky

**Affiliations:** 1 Hospital de Pediatria Garrahan, Endocrinology Service, Buenos Aires, Argentina; 2 National Scientific and Technical Research Council (CONICET), Endocrinology Service, Buenos Aires, Argentina

**Keywords:** 46, XY disorders of sex development, androgen insensitivity syndrome, androgen receptor gene mutations, mosaicism, clinical phenotype

## Abstract

**Objective:**

The aim of this study was the molecular characterization of the *AR* gene as the cause of 46,XY disorder in our population.

**Methods:**

We studied 41, non related, 46,XY disorder of sexual differentiation index cases, having characteristics consistent with androgen insensivity syndrome (AIS). Genomic DNA was isolated from peripheral blood leukocytes of all patients and 25 family members from 17 non-related families.

**Results:**

The *AR* gene analysis revealed an abnormal sequence in 58.5% of the index patients. All of the complete AIS (CAIS) cases were genetically confirmed, while in the partial form (PAIS) a mutation in *AR* was detected in only 13 (43.3%). Molecular studies revealed other affected or carrier relatives in 87% of the index cases. The *AR* mutations were found spread along the whole coding sequence, with a higher prevalence in the ligand binding domain. Nine out of 23 (39%) *AR* mutations were novel. In 17% of patients with detected *AR* mutations, somatic mosaicism was detected in leucocyte DNA. In our cohort, long-term follow up gender dysphoria, raised as male or female, was not found. Finally, in suspected PAIS, the identification of *AR* mutation occurred significantly less than in CAIS patients.

**Conclusion:**

Improved knowledge of the components of the *AR* complex and signaling network might contribute to long term outcome and genetic counseling in AIS patients.

**What is already known on this topic?**

Androgen insensitivity syndromes (AIS) the most frequent known monogenic cause of 46,XY disorder of sexual differentiation. Mutations of variable severity in androgen receptor gene are associated with a wide phenotypic spectrum, ranging from complete AIS to a partial form or a mild form.

**What this study adds?**

Characterization of the clinical phenotype, long term follow up, in particular gender identityand the contribution of the AR gene to the molecular cause of 46,XY disorder of sexual differentiation in a single tertiary pediatric center of Buenos Aires, Argentina are reported. Nine novel AR mutations are described.

## Introduction

The endogenous androgens, testosterone (T) and dihydrotestosterone (DHT), exert their effects via a single intracellular receptor protein, the androgen receptor (*AR*) (1). *AR*-mediated androgen action is essential for normal primary male sexual development before birth and for normal secondary male sexual development around puberty, whereas in females, androgens also participate in sexual development around puberty and in adult female sexual function (2). The *AR* gene is located on the X-chromosome inthe Xq11–12 region and encodes a protein with a molecular mass of approximately 110 kDa. The gene consists of eight coding exons (I to VIII) (3). The *AR* is a transcription factor that belongs to the nuclear receptor subfamily 3, group C, member 4. The protein consists of 920 amino acids that, like other nuclear receptors, is composed of an N-terminal domain (NTD), located on exon 1, a DNA-binding domain (DBD), located on exons 2 and 3 containing two zinc fingers, a hinge region connecting the ligand-binding domain (LBD) to the DBD and a C-terminal LBD, located on exons 4-8 (4).

Androgen insensitivity syndrome (AIS; OMIM 300068) is the most frequent known monogenic cause of 46,XY disorders of sex development (DSD) and is an X-linked recessive condition. Mutations of variable severity in the *AR* gene are associated with a wide phenotypic spectrum, ranging from complete AIS (CAIS) to a partial form (PAIS) or a mild form (MAIS). Patients who present with CAIS exhibit female external genitalia, testes located in the inguinal or abdominal area, and complete breast development with sparse to absent axillary and pubic hair. Patients with PAIS present with a predominantly male phenotype with hypospadias or a predominantly female phenotype with cliteromegaly and/or posterior labial fusion, ambiguous genitalia and variable degrees of gynecomastia at puberty. Patients with MAIS present with normal external male genitalia associated with infertility (5).

The aim of this study was to characterize the clinical phenotype and the contribution of the *AR* gene to the molecular cause of 46,XY DSD in our population.

## Methods

We studied 41 unrelated 46,XY DSD patients with clinical and hormonal characteristics consistent with AIS. CAIS was suspected in 11 of the patients and PAIS in 30. All patients presented with female or ambiguous external genitalia, adequate T production without evidence of steroidogenic blockade and no Müllerian structures evident on abdominal ultrasound. Patients with hormonal determinations previous to gonadal biopsy or gonadectomy presented no biochemical evidence of gonadal dysgenesis and had normal male follicle-stimulating hormone (FSH) levels. In these individuals, the *AR* gene was the first candidate for molecular analysis.

Informed consent for the genetic study was obtained from all of the patients or their parents guardians after full explanation of the purpose and nature of all procedures.

The study was approved by the Independent Ethics Committee “Prof. Dr. J. P. Garrahan Pediatric Hospital” (reference number: 971).

### Hormonal Assays

Serum luteinising hormone (LH) and FSH levels were determined by the IMX systems (Abbott Laboratories, Abbott Park, IL); assay sensitivity was 0.3 IU/L for LH and 0.2 IU/L for FSH; interassay coefficient of variation ranged from 3.1-8.7% for LH and from 3.8-12% for FSH. Serum anti-Müllerian hormone (AMH) levels were determined by ELISA; assay sensitivity was 0.5 pmol/L and assay limit of quantification 1.2 pmol/L serum T was determined by a DPC Immulite^®^ Assay System (Diagnostic Products, Los Angeles, CA); assay sensitivity was 0.17 nmol/L; interassay coefficient of variation ranged from 7.4 to 13%.

### *AR* Gene Mutation Analysis

Genomic DNA was isolated from peripheral blood leukocytes of all patients (41 index cases) and 25 family members from 17 families according to standard procedures. There were seven families in which family members were not available for molecular studies. The entire coding region (exons 1-8) and splice sites in flanking intronic regions of *AR* gene were polymerase chain reaction (PCR) amplified and sequenced by automated analysers (6).

After PCR, the products were assessed by electrophoresis on a 1% agarose gel stained with ethidium bromide and showed a single band with expected size. The PCR products were purified (Qia Quick PCR Purification Kit, Qiagen, Buenos Aires, Argentina) and sequenced using a BigDye Terminator version 3.1 cycle sequencing kit (Applied Biosystems, Buenos Aires, Argentina) on an ABI PRISM^®^ 3130 Genetic Analyzer capillary DNA sequencer (Applied Biosystems, Buenos Aires, Argentina). The primers used for sequencing were the same as those used for PCR. Previously reported intronic mutations were also analysed [Human Gene Mutation Database (HGMD), www.hgmd.cf.ac.uk/]. The nucleotide sequences obtained were compared with those from Genebank accession number: NG_009014.2. Nucleotide changes were reconfirmed in each sample DNA by antisense sequence and resequencing after a new PCR product was produced from the original DNA extract.

### *In silico* Protein Analysis

Nonsense and frameshift mutations which implicate a premature stop codon and a truncated protein were considered deleterious.

The sequence homology-based tool, [Sorting Intolerant from Tolerant (SIFT); http://sift.jcvi.org/], version 2.0.6, the structure-based tool PolyPhen-2 (Polymorphism Phenotyping v2, http://genetics.bwh.harvard.edu/pph2/) and Mutation Taster (http://www.mutationtaster.org/) were used to predict the pathogenicity of the previously undescribed missense variants using default settings. To evaluate the implication of a novel synonymous mutation, we used The Berkeley Drosophila Genome Project (http://www.fruitfly.org/) as a splice site prediction program.

The SIFT algorithm predicts the functional importance of the substitutions based on the alignment of orthologous and/or paralogous protein sequences. The PolyPhen-2 algorithm predicts the functional effects of amino acid changes by considering conservation, physicochemical differences and the proximity of the substitution to the predicted functional domains. Unlike SIFT or PolyPhen which handle only single amino acid substitutions, MutationTaster works on the DNA level and allows insertions and deletions up to 12 base pairs.

The original sequence of the protein was obtained from the Ensembl and UniProt/Swiss-Prot databases.

### Statistical Analysis

This study describes the genotype and clinical phenotype of patients with AIS. A statistical analysis was not necessary.

## Results

In our study *AR* gene analysis revealed an abnormal sequence in 24 individuals (58.5% of total index patients). All of the CAIS cases (n=11) were genetically confirmed, while in PAIS (n=30) a mutation in *AR* was detected in only 13 patients (43.3%).

Family studies were performed in 25 family members from 17 families. The molecular studies and affected family members are shown in Table 1. Molecular studies revealed other affected or carrier relatives in 87% of the index cases. *De novo* *AR* mutations were found in three (P3, P5 and P6) out of 13 mothers analyzed. In two non-related index cases (P12 A and P15), two 46,XY affected siblings raised as female were detected. Interestingly, even though in P12A PAIS was established, normal external female genitalia, in the affected sister, was observed (P12 B). As shown in Table 1, 23 *AR* mutations were detected. The *AR* mutations were found spread along the whole coding sequence, with a higher prevalence in LBD: 8.3% were located in NTD; 16.6% in the DBD; 70.8% in the LBD and 4.3% were gross deletions (7). 

Nine out of 23 (39%; P1, P6, P8, P13, P15, P16, P22, P23 and P24) *AR* mutations were novel. Two novel mutations were located in the NTD domain (P1 and P16). They were both out of frame deletions that ultimately created a nonsense stop codon and premature truncation of the protein. The others, located in the LBD, were:four missense mutations, a nonsense mutation together with a 2bp deletion and a duplication of 7bp that produce a frameshift with a premature stop codon. Three patients (P6, P8 and P16) harboured somatic mosaicisms: a nonsense mutation, a 7bp duplication and a 20bp deletion which result in a truncating frameshift mutation. One missense mutation was located in the DBD. All novel mutations were predicted to be pernicious by all *in silico* tools.

In four individuals (P5, P6, P8 and P16), 17% of AR-mutated gene patients, somatic mosaicism of mutant and wild type alleles was detected in DNA derived from blood leukocytes. 

Of the 17 individuals without a defect in the AR, two patients were finally diagnosed (and genetically confirmed) with 5-alpha reductase deficiency. In the others, diagnosis remains unknown. 

The clinical phenotype and follow-up of the genetically confirmed patients is shown in Supplemental Table 1. Interestingly, during follow-up, no gender dysphoria, including those PAIS patients assigned male or female sex, were observed. Unfortunately, in toddler patients, gender identity could not be evaluated. According to previous reports, very low frequency of gonadal tumors was found. Only in P17 was a Sertoli cell tumor detected (8).

## Discussion

We describe a series of unrelated patients affected by different degrees of AIS. *AR* gene mutations are the main cause of 46,XY DSD. To date, the *AR* gene mutations database (http://www.mcgill.ca/androgendb/) has reported more than 800 different *AR* mutations from patients with AIS. 

In all CAIS cases, *AR* mutations responsible for the phenotype were identified. However, similar to other cohorts, in PAIS phenotype cases, *AR* mutations were identified in only 38%. Overall, in our series of 41 index patients, the *AR* gene proved to be abnormal in 58.5%, confirming the diagnosis. Similarly, Boehmer et al (9) and Audi et al (2) report a frequency of detection of 44-65% which is in line with our results. In contrast de Silva et al (10) and Akcay et al (11) describe cohorts with 15-18% of genetically confirmed AIS. In these studies, the significantly lower percentage of *AR* mutation detection could be due to the presence of overlaps in the clinical presentation of the patients, such as 5-α reductase deficiency or the fact that patients with a T biosynthetic defect were also included. Therefore, it has been proposed that even though AR is essential for virilization, other components of the AR complex and signaling network are required for complete masculinization. It has been suggested that in non-detected cases androgen resistance might be secondary to mutations in the 5’UTR, or other regulatory regions. Moreover, several necessary AR cofactor(s) should also be taken into consideration. Several cofactors, such as coactivators steroid receptor coactivator 1 (SRC1), transcriptional mediators/intermediary factor 2, SRC3 and corepressors nuclear receptor-interacting protein 1, nuclear receptor subfamily 0 group B member 1, are actively involved in the regulation of *AR*-mediated transcription, and might play an important role in AIS etiopathogenesis (12,13,14,15). Interestingly, in order to confirm androgen resistance, Hornig et al (16) developed a DHT-dependent transcriptional induction of the androgen-regulated *APOD* *(apolipoprotein D)* gene in cultured genital fibroblasts (*APOD*-assay). However, the usefulness of this *APOD assay* needs to be confirmed in a large cohort.

Mutations in the *AR* gene are distributed throughout the sequence with a preponderance (70.8%) located in the LBD (17). In our cohort, nine novel AR mutations were found, expanding the mutational spectrum of 46, XY DSD. In three of these novel mutations, located in the LBD, a truncated, significantly reducedor inactive protein was predicted due to a premature stop codon, secondary to gene deletion (P15), gene duplication (P8) or nonsense mutation (P6). The p.Phe726Cys missense mutation located in the LBD was also detected. Interestingly the study of Quigley et al (18) demonstrated by functional assays that a missense mutation in the same position (p.Phe726Leu),caused the disruption of the N/C terminal interaction of the mutated protein. Hence it might be reasonable to suppose that the novel missense mutation found in our cohort might also affect the transactivation activity of the AR, impairing the binding of the ligand to its LBD. The remaining novel mutations, two gene deletions (P1 and P16) located in the NTD domain, result in a truncated protein due to a premature stop codon.

A lack of correlation between genotype and clinical phenotype has previously been reported (19). Interestingly, in siblings of family 12, harbouring p.Asp691del mutation, a clinical variability was evident. A CAIS phenotype was observed in one case, while in the other a PAIS phenotype was observed. Petroli et al’s (20) study showed, in N/C terminal interaction assays, different profiles of the mutant AR protein in response to DHT stimulation, explaining the phenotypic diversity observed in PAIS cases. 

Somatic mosaicism has been reported. Interestingly, even though the patients carried severe *AR* mutations, PAIS clinical phenotype was reported. In these affected patients the *de novo* mutation occurred after the zygote stage and probably very early, during the first few cell divisions. Thus, different proportions of cells containing mutant or wild-type protein are present in various tissues of the same individual explaining the mild phenotype. Similarly, in four patients of our cohort (P5, P6, P8 and P16) a severe mutation was detected but presenting with a PAIS phenotype. It is noteworthy that detection of somatic mosaicism in AR has a great impact for patients with AIS because further virilization is possible after birth and this is an important consideration for genetic counseling (21).

No gender dysphoria was observed in our cohort, even though systematic assessment was not available in all cases.

In contrast to previous reports, in this cohort, AMH serum concentrations during the neonatal period were within the normal male reference range in the only two PAIS cases in whom it was assessed (22,23). *AMH* gene expression in Sertoli cells is inhibited via the AR receptor pathway (24). The lack of *AR* expression in Sertoli cells during mini puberty could explain our findings, suggesting that other as yet unidentified factors might be involved in the regulation of AMH synthesis (25). 

In agreement with previous reports, normal gonadotropin levels were the most frequent finding (26).

### Study Limitations

Even though all *in silico* tools predicted the novel mutations to be damaging for protein structure and function, functional assays should be performed to confirm pathogenicity.

## Conclusion

In summary, we report a series of 41 46,XY DSD index patients in whom AR was the candidate gene. Molecular diagnosis is useful for genetic counseling of the families. However, similar to other series, the percentage of suspected casesin whom an AR mutation was found wasonly around 60%.

Emerging technological advances might contribute to an increase in the accuracy of determining the etiology in suspected AIS cases.

## Figures and Tables

**Table 1:**
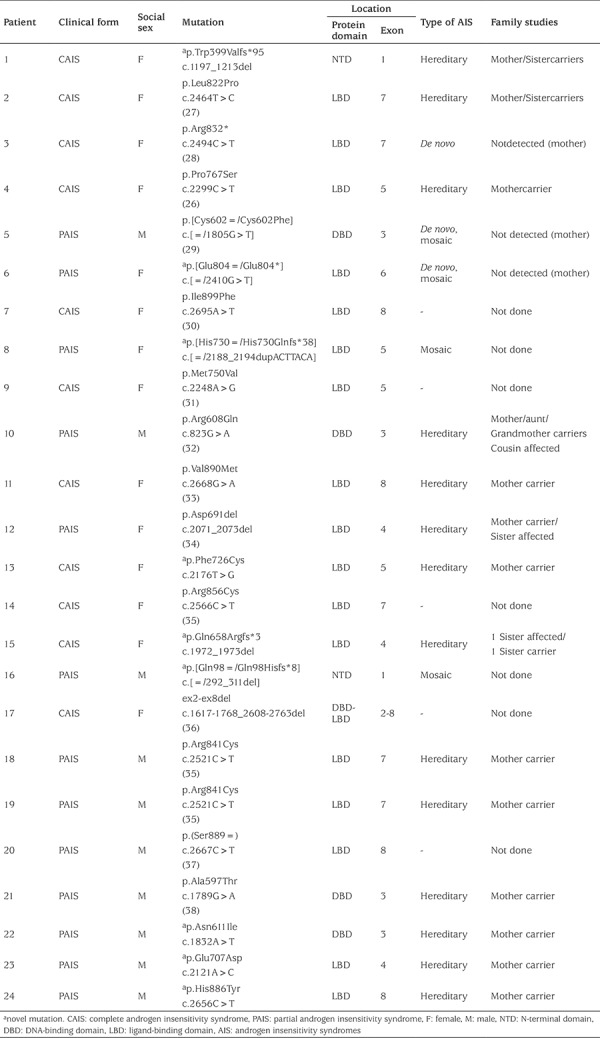
Clinical phenotype, social sex and molecular studies

**Supplemental Table 1:**
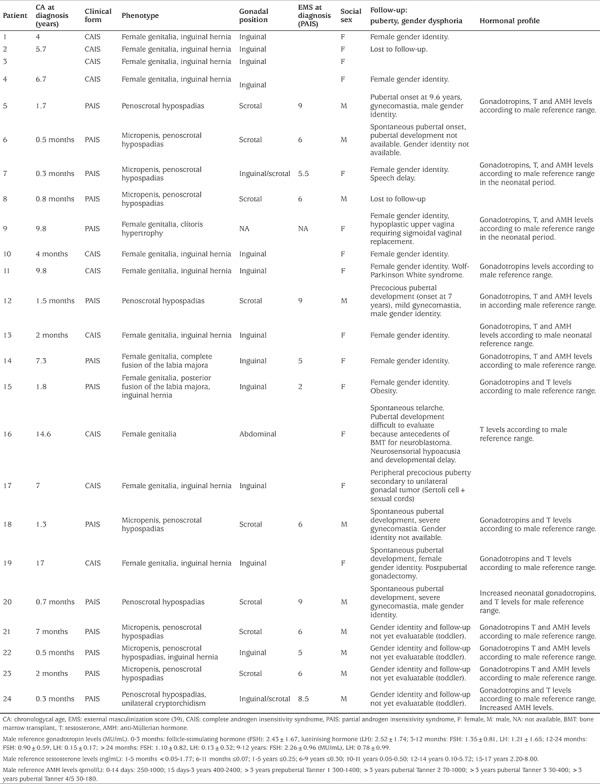
Clinical phenotype at diagnosis and long term follow-up
